# Supplementary material for: Reconfigurable Stochastic neurons based on tin oxide/MoS2 hetero-memristors for simulated annealing and the Boltzmann machine
Source: Nat Commun. 2021 Sep 29;12:5710. doi: 10.1038/s41467-021-26012-5 (PMC8481256; doi:10.1038/s41467-021-26012-5)
Supplement: Supplementary file 1 — Supplementary Information [file 41467_2021_26012_MOESM1_ESM.pdf]

Supplementary Information

Section 1. Optical micrograph of the top-view hetero-memristive device.

As shown in Supplementary Figure 1, the top electrode (TE) is deposited on the top of SnO_x, and V_{TE} is applied to this electrode. The bottom electrode (BE) is deposited on MoS_2 and is grounded. The gate bias V_g is applied to the Si back gate. The current path for this device is from the top electrode to the SnO_x/MoS_2 heterostructure.

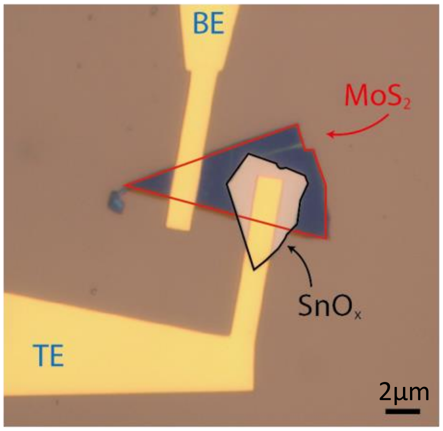

Supplementary Figure 1. Optical micrograph of a fabricated hetero-memristive device.

Section 2. Device Endurance

The device is measured up to 5000 switching cycles. The data is also extrapolated to the 10^6 cycles mark. The set pulse is 5V and the read pulse is 1V. the reset pulse is -5 V and the read pulse is 1V.

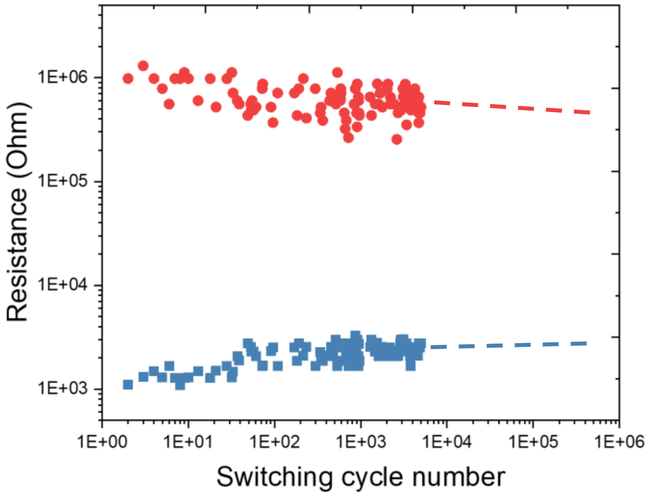

Supplementary Figure 2. Endurance of the device with consecutive switching cycles.

Section 3. c-AFM characterization of conductive filament formation

The conductive atomic force microscopy (c-AFM) measurement was carried out to image the conductive filament in SnO_x. In this test, bias voltage is applied through the AFM tip to form the conductive filament in SnO_x. c-AFM measurement then maps the current in the active region after the filament is set. Supplementary Figure 3 shows the test structure (same structure as the actual device with the material layers in reverse order to allow c-AFM probing) used in the c-AFM measurement. The tip acts as a conductive probe to apply the voltage at the top of MoS_2 layer, thereby setting the device into either the ON state or OFF state. A voltage of -5 V is applied at the AFM tip (20 nm diameter) to set the device and the characteristic I-V curve shown in Supplementary Figure 4a demonstrates the successful set process. After set, a small read voltage (-2 V) is applied to map the current levels. In Supplementary Figure 4b, the green area indicates the region of higher current density (~10 nA), which is the location of the conductive filament.

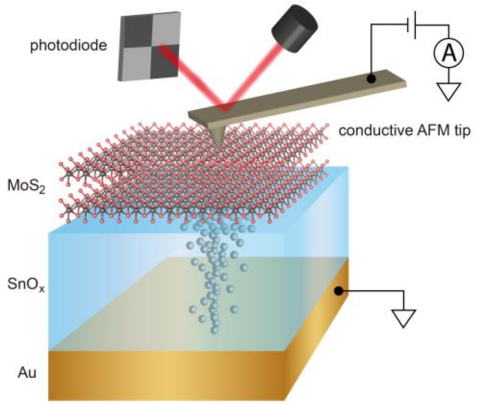

Supplementary Figure 3. Test structure for c-AFM characterization.

The filament diameter is about 20 nm.

During the first switching cycle, a higher applied voltage is needed to form the initial filament as measured in the conductive AFM (c-AFM) characterization (shown in Supplementary Figure 4a, black curve). The forming voltage is around -3.2 V (the voltage is negative due to the reverse device structure used in the c-AFM test (Supplementary Figure 3)). After the initial forming process, the set voltage reduces to around -2.6 V for this test structure, lower than the forming voltage (Supplementary Figure 4a, red curve).

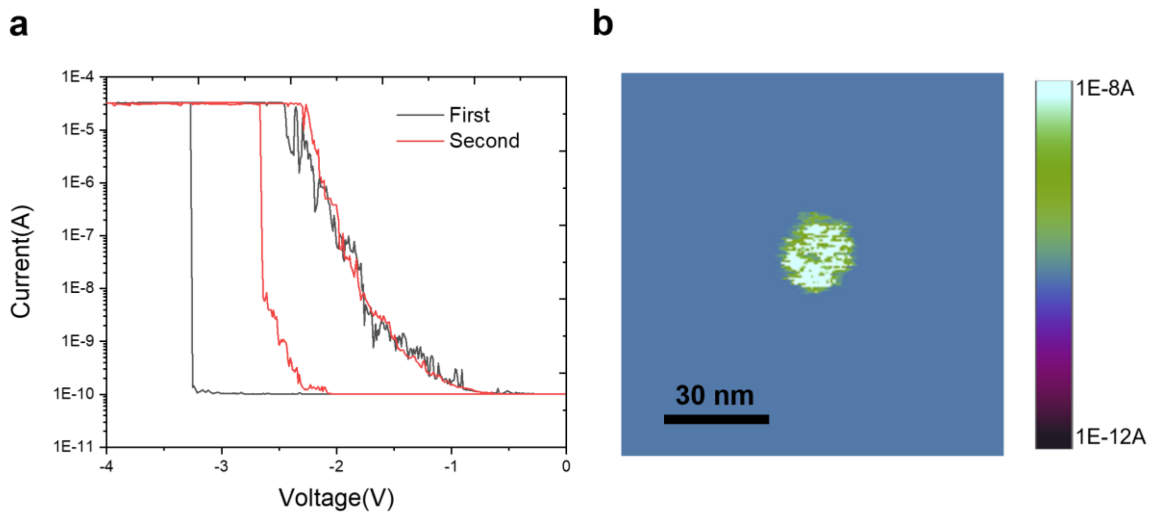

Supplementary Figure 4. **a**, First set curve (represents forming process) and subsequent set curve. **b**, Current maps read by -2V after device is set in the c-AFM characterization. The green (and bright) area shows the position of higher current density.

Section 4. Gate modulation on the hetero-memristive device and the band diagram

The schematic band diagram along the current path formed in the MoS_2 layer in series with the filament in the SnO_x layer is shown in Supplementary Figure 5, where E_F=0 is the Fermi level of GND, $E_{F,TE}$ is the Fermi level of the top electrode, and $F_{n,MoS2}$ is the electron quasi-Fermi level in MoS_2 at the position of the vertical filament contact. The short lines schematically represent the oxygen vacancy levels in the SnO_x layer. The MoS_2 material used in this experiment has multiple layers. Its quasipartical bandgap is $E_g \approx 1.29$eV[1]. The bandgap of SnO_x thin films is adjustable between 3.7 and 4.1eV[2], which is significantly larger than the bandgap of MoS_2. At low V_G, the

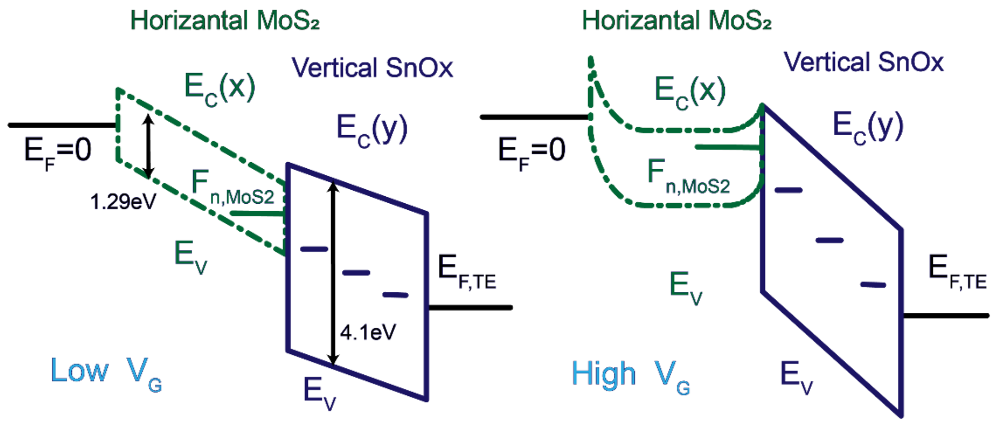

Supplementary Figure 5. Band diagram of SnO_x/MoS_2 hetero-memristive device under low and high V_G.

semiconducting MoS_2 layer has a low carrier density and thicker interface barrier, and as the gate voltage increases, the carrier density in MoS_2 increases and the contact barrier thickness can be reduced by the gate modulation, which results in lower resistance.

Section 5. Switching speed and its effect on the sampling characteristics.

Switching speed in our device is mainly dominated by the voltage that falls on SnO_x. Higher voltage can produce higher electric field and higher current, which facilitates oxygen ion movement and speed up the filament dynamics and increase the switching speed.

Theoretically, the filament formation is controlled by microscopic ionic hopping and ionization process, whose rates can be expressed as, $r = r_0 \exp(-\frac{E_b - d\epsilon}{k_B T})$, where r_0 is a rate constant, E_b is the barrier height, d is the effective hopping distance, ϵ is the electric field along the hopping direction[3]. This equation shows that the speed of filament dynamics is approximately exponentially dependent on the electric field. This has been demonstrated both analytically and experimentally[4,5].

Section 6. Derivation of the probability distribution that the first SET will occur within a small interval Δt at time t.

We define t as the amount of time when the bias voltage is applied on top electrode until the device sets. Ignoring the device reset time, we can repeat the SET process as defined in main text and count the set events as arrivals. We can then define a Poisson process[6] in terms of sequence of inter-arrival times, $X_1, X_2, X_3, \ldots$, which are independent and identically distributed (IID) random variables. The counting random variable $N(0,t)$ is defined as the number of sets that happens in the time interval $(0,t]$. $N(t) = 0$ means the device does not set within $(0,t]$. $N(t) = k$ means the device succeeds to set k times within $(0,t]$.

For Poisson distribution, $P(N(t) = k) = \frac{e^{-\frac{t}{\tau}}\left(\frac{t}{\tau}\right)^k}{k!}$. The probability that the first set will occur within an infinitesimal interval Δt at time t can be derived as:

$$P = P(N(0, t - \Delta t) = 0, N(t - \Delta t, t) = 1)$$

which means at $(0, t-\Delta t)$ there is no set event, and the first set happens at $(t-\Delta t, t)$.

$$P = P(N(0, t-\Delta t) = 0) \cdot P(N(t-\Delta t, t) = 1)$$

$$= e^{-\frac{t-\Delta t}{\tau}} \cdot e^{-\frac{\Delta t}{\tau}} \cdot \frac{\Delta t}{\tau}$$

$$P = e^{-\frac{t}{\tau}} \cdot \frac{\Delta t}{\tau} \quad (1)$$

Here, τ is average time needed for the device to set.

Section 7. Derivation of the probability distribution that the device will set successfully in a SET process within t_0 seconds, i.e. $P_{\mathrm{ss,t<t_0}}$.

In our device, the SET process is determined by filament formation through ion hopping. In the stochastic ion hopping transport, the average hopping rate is exponentially dependent on the barrier height, which can be reduced by applying a voltage. Therefore, τ is exponentially dependent on the bias and gate voltage, and it can be described as

$$\tau(V_{\mathrm{TE}}) = \tau_0 e^{-\frac{V_{\mathrm{TE}}}{V_{\mathrm{g}}}}, \quad (2)$$

where τ_0 is fitting parameters in equation (2).

In each trial, the device either sets or not within t_0 seconds, thus $P_{\mathrm{ss},t<t_0}$ can be derived analytically as follows:

$$P_{\mathrm{ss},t<t_0} = 1 - P(N(t_0) = 0)$$

$$P_{\mathrm{ss},t<t_0} = 1 - e^{-\frac{t_0}{\tau}}$$

Replace τ with equation (2),

$$P_{\mathrm{ss},t<t_0} = 1 - e^{-\frac{t_0}{\tau_0 e^{-\frac{V_{\mathrm{TE}}-V_{\mathrm{TE0}}}{V_{\mathrm{g}}}}}} = 1 - e^{-\frac{t_0}{\tau_0} e^{\frac{V_{\mathrm{TE}}-V_{\mathrm{TE0}}}{V_{\mathrm{g}}}}}$$

$$P_{\mathrm{ss},t<t_0} \overset{\mathrm{def}}{=} 1 - e^{-\beta e^{\alpha(V_{\mathrm{TE}}-V_{\mathrm{TE0}})}} \quad (3)$$

where $\beta = t_0/\tau_0$ and $\alpha = 1/V_{\mathrm{g}}$ can be treated as fitting parameters that are related to material and

device structure. In this work, we mainly discuss the condition of time limit $t_0 = 2$.

Section 8. Asymptotic behaviors of the probability function

The probability function of the stochastic device characteristics is described in equation (3). This function has these limits, i.e, $P_{\mathrm{ss},t<2\mathrm{s}}(V_{\mathrm{TE}} \to -\infty) = 0$ and $P_{\mathrm{ss},t<2\mathrm{s}}(V_{\mathrm{TE}} \to +\infty) = 1$. For a sufficiently small V_{TE} satisfying $\beta e^{\alpha(V_{\mathrm{TE}})} < 0.1$, equation (3) is simplified by using the first order Taylor expansion,

$$P_{\mathrm{ss},t<2s} \approx \beta e^{\alpha(V_{\mathrm{TE}}-V_{\mathrm{TE0}})} \approx \frac{1}{1+\left(\beta e^{\alpha(V_{\mathrm{TE}})}\right)^{-1}} = \frac{1}{1+e^{-\frac{V_{\mathrm{TE}}-V_{\mathrm{TE0}}}{T_{\mathrm{eff}}}}}, \quad (4)$$

where $V_{\mathrm{TE0}} = In(\beta)/\alpha$ is the 50% probability bias voltage point, and $T_{\mathrm{eff}} = 1/a$ is the effective temperature.

For a sufficiently large V_{TE} satisfying $\beta e^{\alpha(V_{\mathrm{TE}})} > 10$, equation (3) is simplified to

$$P_{\mathrm{ss},t<2s} \approx \frac{1}{1+e^{-\beta e^{\alpha(V_{\mathrm{TE}}-V_{\mathrm{TE0}})}}}, \quad (5)$$

which indicates that the function asymptotically approaches to the Fermi-Dirac distribution function or sigmoid function. This feature makes the tin oxide/MoS_2 device perfectly suit for realize the exponential-class distribution sampling, especially for the Boltzmann machine whose probability of turning on a unit *i* has the similar form:

$$P_{s_i=1} = \frac{1}{1+e^{-z_i}}, \quad (6)$$

where z_i is the total input of the Boltzmann machine at unit *i*.

Section 9. Dependence of the “temperature” effect on applied gate voltage

A behavioral model is developed to understand the dependence of the effective “temperature” on gate voltages. The device is modeled as a memristor in serial combination with a MoS_2 layer, whose resistance is modulated by the gate voltage.

The resistance of MoS_2 layer is modulated by gate voltages as

$$R_{\mathrm{MS}}(V_{\mathrm{g}}) = \frac{R_{\mathrm{c}}}{V_{\mathrm{g}}-V_{\mathrm{T}}}, \quad (7)$$

where R_c and V_T are constants.

The voltage on the switching layer is

$$V_{\mathrm{SW}}(V_{\mathrm{g}}) = \frac{R_{\mathrm{SW}}}{R_{\mathrm{SW}}+R_{\mathrm{MS}}(V_{\mathrm{g}})} V_{\mathrm{TE}}, \tag{8}$$

where R_{SW} is the average resistance of the switching layer.

As a result, the gate dependent "temperature" effect of the device can be expressed as

$$T_{\mathrm{eff}}(V_{\mathrm{g}}) = T_{\mathrm{V0}} \left[\frac{R_{\mathrm{SW}}+R_{\mathrm{MS}}(V_{\mathrm{g}})}{R_{\mathrm{SW}}}\right] = T_{\mathrm{V0}} \left[1 + \frac{Z}{(V_{\mathrm{g}}-V_{\mathrm{T}})}\right], \tag{9}$$

where $Z = R_{\mathrm{c}}/R_{\mathrm{SW}}$, and T_{V0} are constants.

Section 10. Simulated BM results

The simulation of the Boltzmann machine operation was conducted, and the simulated results are shown in Supplementary Figure 6. Due to the stochastic nature of BM, we ran the simulation of BM for 50 times and plotted the averaged energy evolution under four "temperature cooling" strategies. The plot indicates the effect of temperature strategy on the optimization process of the BM, which matches well with the experimental results in Figure 4d.

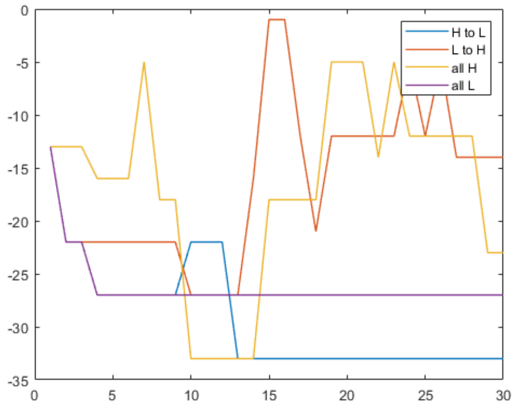

Supplementary Figure 6. The simulated result of energy evolution in BM under 4 different strategies.

Reference

1 Kobayashi, K. & Yamauchi, J. Electronic structure and scanning-tunneling-microscopy image of molybdenum dichalcogenide surfaces. *Physical Review B* **51**, 17085 (1995).

2 Ali, F. *et al.* Tuning of oxygen vacancy in sputter-deposited SnOx films for enhancing the performance of perovskite solar cells. *ChemSusChem* **11**, 3096-3103 (2018).

3 Guy, J. *et al.* in *2014 IEEE International Electron Devices Meeting.* 6.5. 1-6.5. 4 (IEEE).

4 Ielmini, D. Modeling the universal set/reset characteristics of bipolar RRAM by field-and temperature-driven filament growth. *IEEE Transactions on Electron Devices* **58**, 4309-4317 (2011).

5 Zhang, H. *et al.* Gd-doping effect on performance of HfO 2 based resistive switching memory devices using implantation approach. *Applied Physics Letters* **98**, 042105 (2011).

6 Grimmett, G. & Stirzaker, D. *Probability and random processes*. (Oxford university press, 2020).
